# Whole transcriptome analysis reveals correlation of long noncoding RNA *ZEB1-AS1* with invasive profile in melanoma

**DOI:** 10.1038/s41598-019-47363-6

**Published:** 2019-08-05

**Authors:** Ádamo Davi Diógenes Siena, Jéssica Rodrigues Plaça, Luiza Ferreira Araújo, Isabela Ichihara de Barros, Kamila Peronni, Greice Molfetta, Carlos Alberto Oliveira de Biagi, Enilza Maria Espreafico, Josane Freitas Sousa, Wilson Araújo Silva

**Affiliations:** 1 Department of Genetics at Ribeirão Preto Medical School, University of São Paulo, Ribeirão Preto, Brazil; 2 Department of Cellular and Molecular Biology at Ribeirão Preto Medical School, University of São Paulo, Ribeirão Preto, Brazil; 3 Center for Cell-Based Therapy (CEPID/FAPESP); National institute of Science and Technology in Stem Cell and Cell Therapy (INCTC/CNPq), Regional Blood Center of Ribeirão Preto, Ribeirão Preto, Brazil; 4 Center for Integrative Systems Biology (CISBi) – NAP/USP, Ribeirão Preto, Brazil; 5 Institute of Biological Sciences, Federal University of Para, Belem, Brazil; 6 Center for Medical Genomics, HCFMRP/USP, Ribeirão Preto, Brazil

**Keywords:** Melanoma, Gene expression, Transcriptomics, Long non-coding RNAs

## Abstract

Melanoma is the deadliest form of skin cancer, and little is known about the impact of deregulated expression of long noncoding RNAs (lncRNAs) in the progression of this cancer. In this study, we explored RNA-Seq data to search for lncRNAs associated with melanoma progression. We found distinct lncRNA gene expression patterns across melanocytes, primary and metastatic melanoma cells. Also, we observed upregulation of the lncRNA *ZEB1-AS1* (ZEB1 antisense RNA 1) in melanoma cell lines. Data analysis from The Cancer Genome Atlas (TCGA) confirmed higher *ZEB1-AS1* expression in metastatic melanoma and its association with hotspot mutations in *BRAF* (B-Raf proto-oncogene, serine/threonine kinase) gene and *RAS* family genes. In addition, a positive correlation between *ZEB1-AS1* and *ZEB1* (zinc finger E-box binding homeobox 1) gene expression was verified in primary and metastatic melanomas. Using gene expression signatures indicative of invasive or proliferative phenotypes, we found an association between *ZEB1-AS1* upregulation and a transcriptional profile for invasiveness. Enrichment analysis of correlated genes demonstrated cancer genes and pathways associated with *ZEB1-AS1*. We suggest that the lncRNA *ZEB1-AS1* could function by activating *ZEB1* gene expression, thereby influencing invasiveness and phenotype switching in melanoma, an epithelial-to-mesenchymal transition (EMT)-like process, which the *ZEB1* gene has an essential role.

## Introduction

Melanoma is the most lethal form of skin cancer. Although it comprises only 5% of skin cancers, it corresponds to 80% of patient deaths^1^. While trends for most cancers have been declining worldwide, the incidence of melanoma has risen in the last decades^2^. Melanoma progression strongly impacts on patient lifespan, with the 5-year survival rate decreasing from 98% of patients with local melanoma to only 16% of patients with distant melanoma^3,4^. As for other cancers, metastasis is the leading cause of melanoma deaths, making detection of early disease, when curative resection is possible, considered the most effective treatment.

Currently, the tumour, lymph node, metastasis (TNM) staging system does not provide an accurate prognosis for melanoma^3^, reflecting the need to identify biomarkers^5^ with better diagnostic and staging and predictive values. Furthermore, the identification of molecules and mechanisms to confront melanoma development has improved rapidly in the last decades and could in the future provide a new source of treatment strategies. Recent genomic studies have identified a functionally diverse class of noncoding RNAs, defined as long noncoding RNAs (lncRNAs)^6^, which has been associated with gene regulatory functions. These molecules are transcripts longer than 200 nucleotides lacking protein-coding capacity. They are versatile molecules involved in gene expression regulation by diverse interactions with DNA, RNA and/or proteins^7,8^. Studies on lncRNAs expression patterns and function have demonstrated enough evidence of their key role in developmental processes^9^ and diseases^10^, including many cancers^11^. In melanoma, lncRNAs have recently been associated with important biological features, such as proliferation^12–14^, invasion^15,16^ and apoptosis^17,18^; however, details of their influence on melanoma development are still missing.

Previous findings of our laboratory demonstrated the influence of the lncRNA *HOTAIR* (HOX transcript antisense RNA) in stemness and epithelial-to-mesenchymal transition (EMT) in cancer^19^. The EMT process is a highly conserved critical step for embryogenesis and melanocyte lineage differentiation. Importantly, reactivation of EMT in many epithelial cancers is common and implicated in the loss of cell polarity and cell-to-cell adhesion, cytoskeleton reorganization and gaining of mesenchymal-like morphology, including the ability of migration^20,21^. A similar process has been observed in melanoma progression, in which melanocytic cells can reversibly change their status towards a more mesenchymal-like state^22,23^. This melanoma “phenotype switching” model is concordant with gene expression profiling studies, which demonstrated two alternating phenotypic profiles switching from a rapidly proliferative/low invasive condition to slower proliferative/highly migratory with high invasive potential^24^. Remarkably, proliferative melanoma samples express high levels of *MITF* (melanocyte inducing transcription factor), *SOX10* (SRY-box 10) and *PAX3* (paired box 3) genes^22,25,26^. In contrast, samples with invasive phenotype demonstrate low levels of MITF and high expression of *ZEB1* and *TGFB1* (transforming growth factor, beta 1) pathway genes^27,28^. Recently, it was confirmed that the melanoma proliferative phenotype is regulated by *SOX10*/*MITF* and that *AP1*/TEADs are regulators of the invasive phenotype^29^. Although several protein-coding genes involved in melanoma phenotype switching have been characterized, the role of noncoding genes in this process remains poorly understood.

Herein, we performed RNA sequencing (RNA-Seq) and downloaded public data to search for lncRNAs associated with melanoma progression. Differential gene expression analysis revealed lncRNA *ZEB1-AS1* upregulation in melanoma in comparison to normal melanocytes. Importantly, significantly higher levels of *ZEB1-AS1* were detected in metastatic melanoma when compared with primary melanoma. Using GSVA (Gene Set Variation Analysis), we found a significant correlation between *ZEB1-AS1* expression and a melanoma invasive transcriptional signature. Our study provides insights into the *ZEB1-AS1* relationship with melanoma progression and suggests its contribution to *ZEB1* regulation, which in turn influences melanoma phenotype switching.

## Results

### Overview of RNA-Seq data from melanocytes and melanoma cell lines

**Figure 1:**
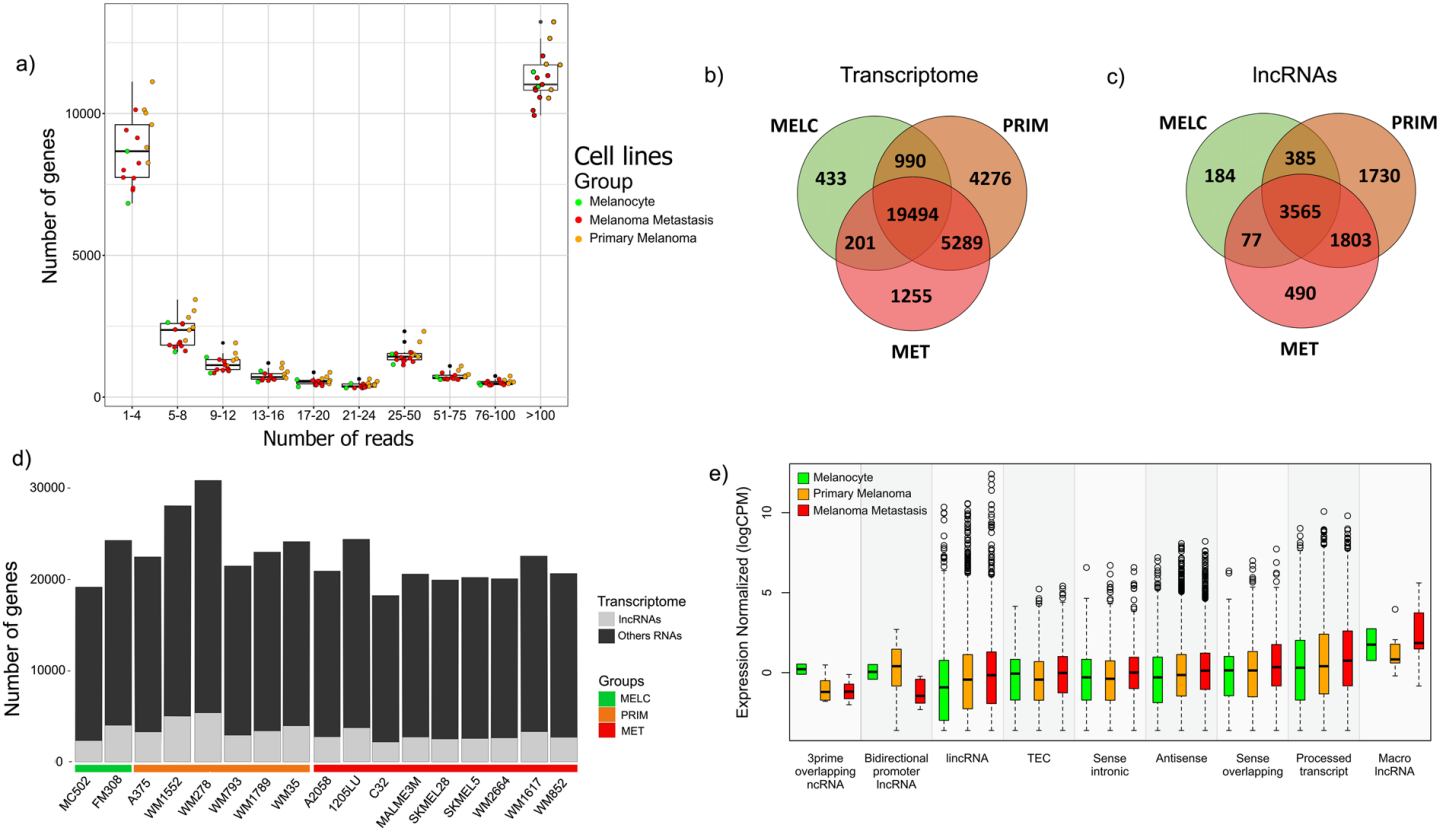
Overview of transcriptome data analysis**. (a**) Boxplot showing the number of all genes detected within regular range of mapped count reads in assembled RNA-Seq data. (**b)** Venn diagram showing transcriptome common and exclusive expressed genes (with mapped reads ≥5) in melanocytes (433 genes), primary melanoma (4276 genes) and metastatic melanoma (1255 genes) cell lines. (**c)** Venn diagram showing only shared and exclusive lncRNAs expressed (with mapped reads ≥5) in melanocytes (184 lncRNAs genes), primary melanoma (1730 lncRNAs genes) and in metastatic melanoma (490) cell lines. (**d)** Characterization of transcriptome composition showing lncRNAs and other genes expressed (with mapped reads ≥5) in melanocytes, primary and metastatic melanoma cell lines. (**e)** Boxplot showing lncRNA biotypes according to GENCODE classification in melanoma progression groups (only considering genes with mapped reads ≥5). Middle lines in boxplot represent median.

We performed RNA-Seq in melanocytic cell lines (melanocytes = 1, primary melanoma = 5, and metastatic melanoma = 3). Subsequently, we downloaded raw public data (melanocyte = 1, primary melanoma = 1, and metastatic melanoma = 6) from a previous study^30^ and assembled all data into a single dataset for subsequent genome mapping and analysis. We obtained read mapping efficiency of 64,39% on average (Supplementary Table S1). Figure 1a shows the number of genes in the human transcriptome per range of mapped reads from the dataset. To avoid non-biological overrepresentation of reads detected and to allow further transcriptome characterization, we only considered a gene being expressed when it presented at least five mapped reads, as previously applied by Tarazona *et al*.^31^. As expected, we detected the expression of a lower number of lncRNAs in comparison to protein-coding genes in the total transcriptome for each cell line (Fig. 1d). When exploring data for exclusive and common transcripts, the highest number of genes expressed exclusively in a group was found in primary melanoma cell lines (PRIM), which presented a total of 4276 genes, of which 1730 were lncRNA genes. Transcripts exclusively expressed in melanocyte cell lines (MELC) represented a total of 433 genes, of which 184 were lncRNA genes. Besides, metastatic melanoma cell lines (MET) exclusive transcripts represented a total of 1255 genes, of which 490 were lncRNAs genes (Fig. 1b,c). According to the GENCODE lncRNA biotypes classification, the two predominant categories in our dataset were antisense (n = 976) and long intergenic non-coding RNAs – lincRNAs (n = 972) genes. Although being the most frequently detected, the median expression level of lncRNAs in those two biotypes categories were not higher than that from lncRNAs in other categories (Fig. 1e).

### Differential gene expression analysis between melanocytes and primary and metastatic melanoma cell lines

**Figure 2:**
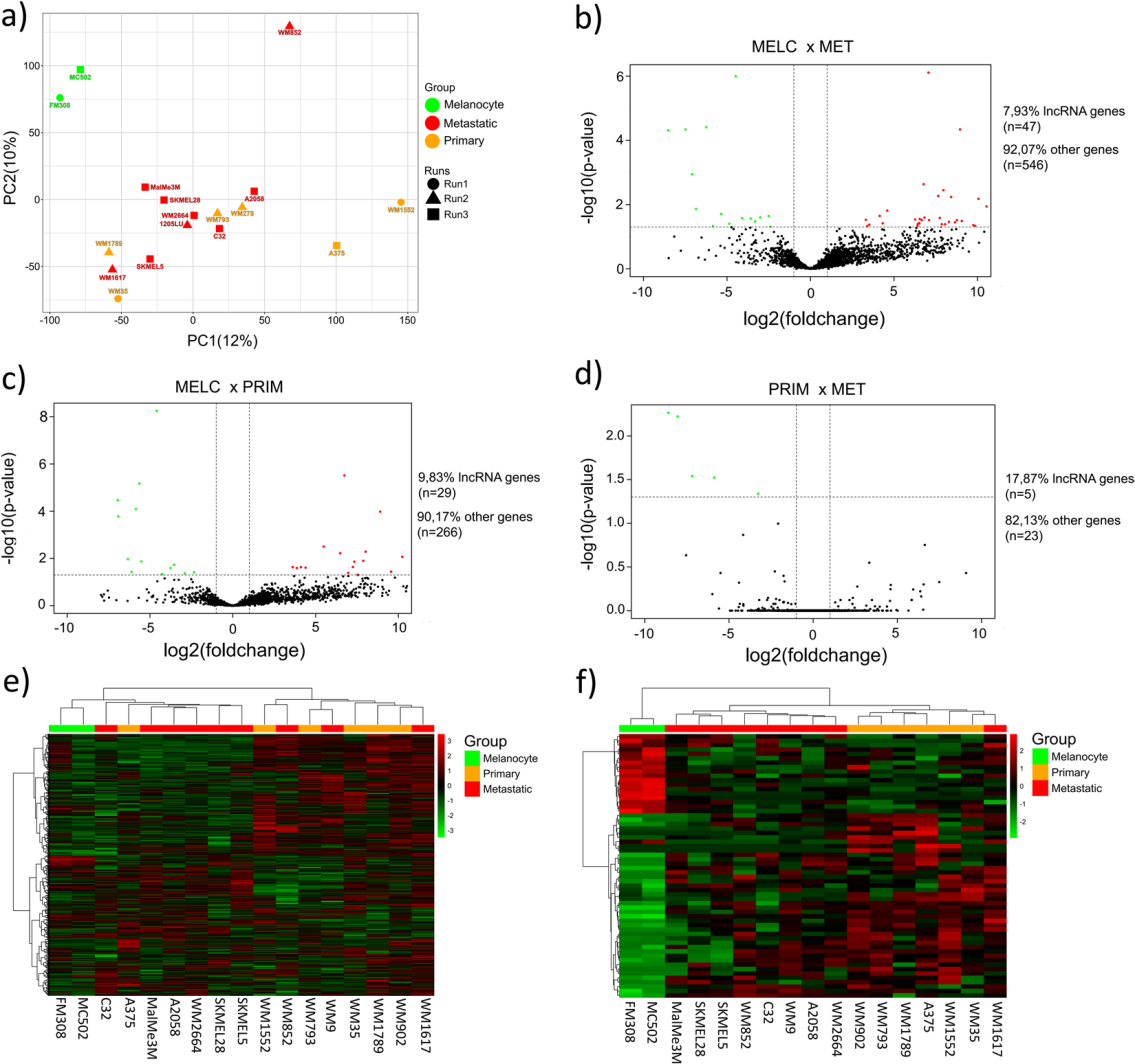
PCA and deregulated expressed genes in melanocyte and melanoma. (**a)** PCA plot showing disparities of melanocytes and melanoma cell lines. Each colour represents a cell line group and shapes represent different runs from the two RNA-Seq data sources. (**b)** Volcano plot showing deregulated lncRNAs in MELC × MET, including upregulated (red dots) and downregulated (green dots) lncRNAs in metastatic melanoma (MET) in comparison with melanocyte (MELC). (**c)** Volcano plot showing deregulated lncRNAs in MELC × PRIM, including upregulated (red dots) and downregulated (green dots) lncRNAs in primary melanoma (PRIM) in comparison with melanocyte (MELC). (**d)** Volcano plot showing deregulated lncRNAs in PRIM × MET, with only downregulated lncRNAs (green dots) in metastatic melanoma (MET) in comparison with primary melanoma (PRIM). (**e,f**) Hierarchical clusterization using Euclidean distance of all differentially expressed genes and considering only differentially expressed lncRNA genes, respectively.

For data quality control and to check cell lines disparities, we performed Principal Component Analysis (PCA). We detected two major clusters distinct from each other, which consists of melanoma (including primary and metastatic) cell lines and melanocyte cell lines (Fig. 2a). We next evaluated differentially expressed genes to detect genes deregulated at different melanoma stages. The analysis comparing melanocytes and metastatic melanoma cell lines (MELC × MET) presented 593 differentially expressed genes, of which 47 were lncRNA genes, including 32 upregulated and 15 downregulated in metastatic melanoma (Fig. 2b). The comparison between melanocytes and primary tumour melanoma cell lines (MELC × PRIM) revealed 295 differentially expressed genes, of which 29 were lncRNA genes, including 16 upregulated and 13 downregulated in primary tumour melanoma cell lines (Fig. 2c). Analysis of primary tumour versus metastatic melanoma cell lines (PRIM × MET) presented 28 differentially expressed genes including five lncRNA genes, all of them downregulated in the metastatic stage (Fig. 2d). We further analyzed expression patterns of differentially expressed genes by unsupervised hierarchical clusterization. We found that melanocytes always clustered together in a distinct manner away from melanoma samples, based either on the total transcriptome (Fig. 2e) or only on the lncRNA gene set (Fig. 2f). However, better separation between the primary and metastatic melanoma cell lines was achieved when performing clusterization based on the lncRNA gene set only.

### The lncRNA *ZEB1-AS1* is upregulated in both primary and metastatic melanoma cell lines in comparison to melanocytes

To further investigate deregulated lncRNA genes, we then selected top upregulated lncRNAs genes according to their Fold Change (FC) values. Pursuing the characterization of lncRNAs potentially associated with both melanoma development and metastatic spread, we prioritised candidates that were deregulated in both comparisons (MELC × PRIM and MELC × MET). The lncRNA with the highest differential expression in both comparisons (MELC × PRIM and MELC × MET) was the recently characterized melanoma-specific lncRNA *SAMMSON*^18^. Similarly, we found the frequently cancer deregulated lncRNA *H19*^32^ amongst the top differentially expressed lncRNAs in our comparisons (see Tables 1 and 2). The third lncRNA most upregulated in primary melanoma cell lines in comparison to melanocytes was *ZEB1-AS1*, which is located in the opposite strand of gene *ZEB1*. Importantly, the lncRNA *ZEB1-AS1* was also amongst the top five lncRNAs upregulated in metastatic melanoma cell lines when compared to melanocytes. The *ZEB1-AS1* persistence as a deregulated lncRNA in different comparisons, as well as the fact that *ZEB1* gene (that we also found upregulated in melanoma cell lines in comparison to melanocytes) is known to contribute to EMT and cancer progression, lead us to prioritize *ZEB1-AS1* for further analysis. Interestingly, in the melanoma cell lines, our analysis revealed a robust positive correlation between *ZEB1-AS1* and *ZEB1* expression levels (Supplementary Fig. S1).

**Table 1:** Top 10 lncRNAs upregulated in primary melanoma cell lines in comparison to melanocytes.

GENE ID	GENE NAME	LOGFC	ADJ. *P*-VALUE	GENE TYPE
ENSG00000240405.5	*SAMMSON*	10,23	5,93E-05	lincRNA
ENSG00000130600.16	*H19*	9,56	4,76E-04	processed transcript
ENSG00000237036.4	*ZEB1-AS1*	8,91	1,48E-07	antisense
ENSG00000268812.3	*RP1-102K2.8*	8,02	2,81E-05	antisense
ENSG00000271857.1	*RP1-244F24.1*	7,88	1,02E-04	antisense
ENSG00000220891.1	*LL22NC03-63E9.3*	7,55	7,64E-04	antisense
ENSG00000231528.2	*FAM225A*	7,34	1,18E-04	lincRNA
ENSG00000257497.2	*RP11-585P4.5*	7,25	2,31E-04	antisense
ENSG00000272872.1	*LL22NC03-N14H11.1*	6,97	5,74E-04	sense intronic
ENSG00000206195.10	*DUXAP8*	6,74	2,83E-09	processed transcript

**Table 2:** Top 10 lncRNAs upregulated in metastatic melanoma cell lines in comparison to melanocytes.

GENE ID	GENE NAME	LOGFC	ADJ. *P*-VALUE	GENE TYPE
ENSG00000240405.5	*SAMMSON*	10,06	6,60E-05	lincRNA
ENSG00000272068.1	*RP11-284F21.9*	10,54	1,42E-04	lincRNA
ENSG00000260604.2	*RP1-140K8.5*	9,86	1,36E-03	lincRNA
ENSG00000244300.2	*GATA2-AS1*	9,75	1,26E-03	antisense
ENSG00000130600.16	*H19*	9,06	7,44E-04	processed transcript
ENSG00000237036.4	*ZEB1-AS1*	8,95	8,68E-08	antisense
ENSG00000259153.1	*RP6-65G23.3*	8,76	6,79E-04	lincRNA
ENSG00000282057.1	*RP4-621F18.2*	8,65	8,85E-04	lincRNA
ENSG00000272502.1	*RP11-713M15.2*	8,42	5,63E-05	antisense
ENSG00000204860.4	*FAM201A*	8,29	8,24E-04	antisense

### Expression levels of lncRNA ZEB1-AS1 and ZEB1 gene are positively correlated in melanoma tumours and associated with frequent melanoma mutations

**Figure 3:**
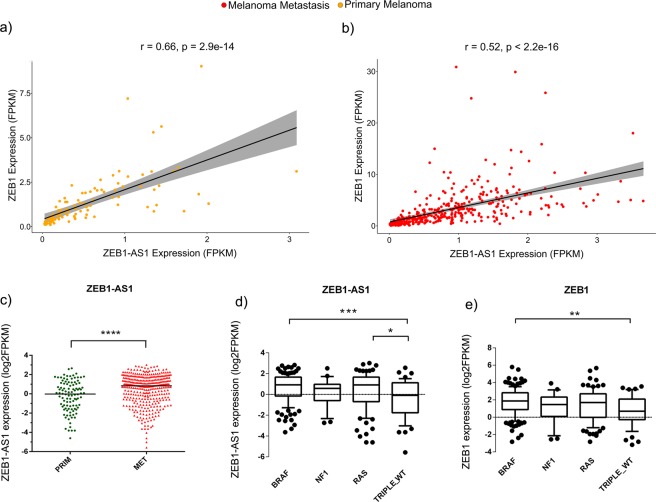
Analysis of *ZEB1* and *ZEB1-AS1* gene expression in TCGA melanoma samples. (**a,b)** High positive correlation between *ZEB1* and *ZEB1-AS1* expression in primary melanoma and metastatic melanoma samples, respectively. Analysis from Pearson’s statistical test. Grey band represents the confidence interval. (**c**) Graph showing higher expression of *ZEB1-AS1* in metastatic melanoma compared to primary melanoma samples from SKCM TCGA. T-test analysis was used. (**d,e)** Boxplot showing expression levels of *ZEB1-AS1* or *ZEB1*, respectively, across different mutational groups of melanoma. Statistics based on ANOVA test. *P*-values statistical significance labels: *P ≤ 0.05, **P ≤ 0.01, ***P ≤ 0.001, ****P ≤ 0.0001.

According to normal tissue data available in the GTEx (Genotype-Tissue Expression) database^33^, *ZEB1-AS1* is a ubiquitously expressed lncRNA, with a tissue distribution pattern similar to *ZEB1* transcription factor (Supplementary Figs S2 and S3). We then evaluated the *ZEB1-AS1* expression in cutaneous melanoma samples (Skin Cutaneous Melanoma - SKCM) from the TCGA data. *ZEB-AS1* expression showed a strong positive correlation with *ZEB1* mRNA in both primary melanomas (Fig. 3a, r = 0.66 and *P-*value < 2.9E-14) and metastatic melanoma (Fig. 3b, r = 0.52 and *P-*value < 2.2E-16) samples. Although melanoma presented lower levels of *ZEB1-AS1* expression in comparison to other tumours (Supplementary Fig. S4), significantly higher levels of *ZEB1-AS1* (*P*-value < 0.0001) were found in metastasis in comparison to primary melanomas (Fig. 3c). Also, we evaluated the molecular subtypes defined by the TCGA network analysis^34^. Melanoma samples carrying *BRAF* or *RAS* mutations presented significantly higher expression levels of *ZEB1-AS1* (*P*-value < 0.001 and *P*-value < 0.05, respectively) when compared to the Triple Wild-Type group (Fig. 3d). Comparatively, *ZEB1* gene demonstrated higher expression (*P*-value < 0.001) only in the *BRAF* group (Fig. 3e).

### Association between lncRNA ZEB1-AS1 gene expression with melanoma invasive phenotype

**Figure 4:**
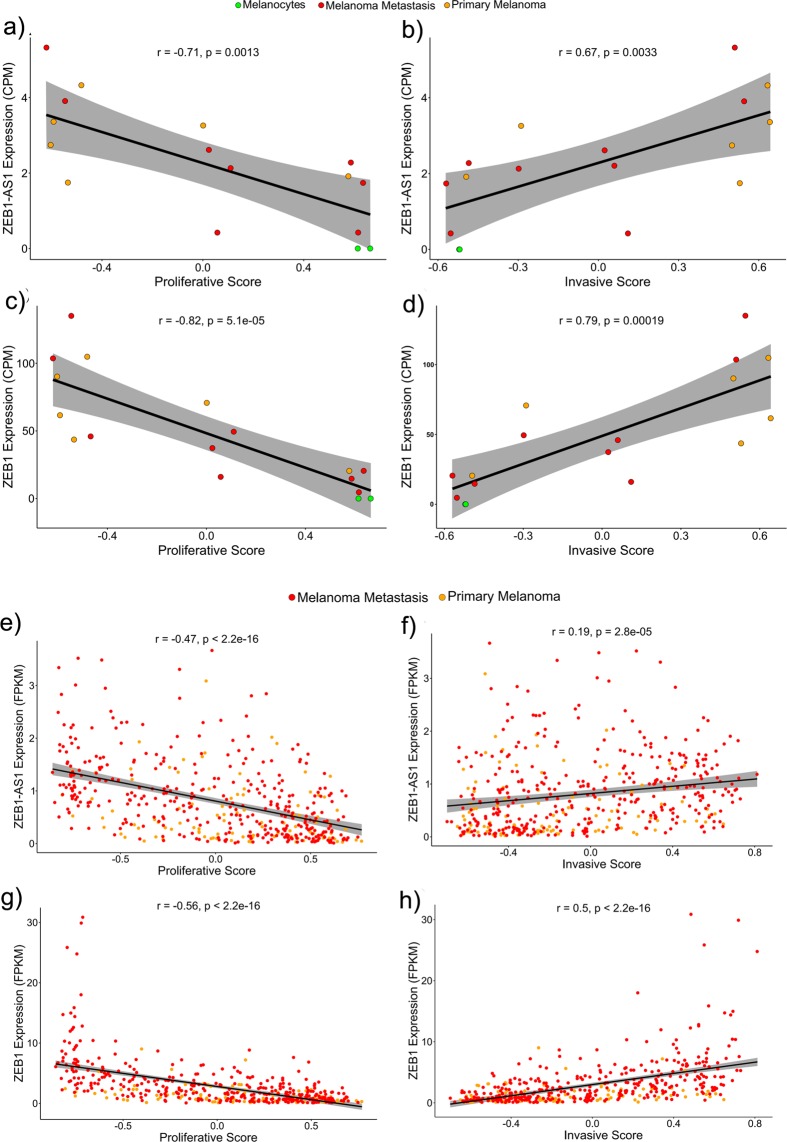
*ZEB1* and *ZEB1-AS1* association with the invasive phenotype. **(a,c)** Correlation plots showing a negative correlation between *ZEB1-AS1* or *ZEB1* expression levels with melanocytic cell lines proliferative score, respectively. (**b,d)** Correlation plots showing a positive correlation of *ZEB1-AS1* or *ZEB1* expression levels with melanocytic cell lines invasive score, respectively. (**e,g)** Correlation plots showing a negative correlation between *ZEB1-AS1* or *ZEB1* expression levels with proliferative score in SKCM TCGA samples, respectively. (**f,h**) Correlation plots showing a positive correlation between *ZEB1-AS1* or *ZEB1* expression levels with an invasive score in SKCM TCGA samples, respectively. Grey band corresponds to the confidence interval.

It has been proposed that during tumour progression melanoma cells switch between a proliferative and an invasive state^35^. Specific gene expression signatures had been associated with each one of these phenotypic states^22^. Using GSVA and the proliferative or the invasive gene signatures we scored each melanoma cell line and each of the tumours sample from the TCGA database for both phenotypic states, based on their expression profile (RNA-Seq data). Then, we evaluated the correlation between *ZEB1-AS1* or *ZEB1* expression levels and the proliferative or invasive scores. Our results revealed that in melanoma cell lines, *ZEB1-AS1* expression presents a robust positive correlation with the invasive score (Fig. 4b, r = 0.67, and *P* = 0.0033) and is inversely correlated with the proliferative score (Fig. 4a, r = −0.71, and *P* = 0.0013). Similar results were observed for *ZEB1* gene expression, with even higher positive correlation with the invasive score (Fig. 4d, r = 0.79, and *P* = 0.00019) and higher negative correlation with the proliferative score (Fig. 4c, r = −0.82, and *P* = 5.1E-05). It is interesting to note that melanocytes, which have the lowest expression levels of *ZEB1* and *ZEB1-AS1*, presented the highest proliferative score and the lowest invasive score values when compared to other melanoma cell lines. Likewise, analysis in the TCGA data set showed *ZEB1-AS1* and *ZEB1* expressions positively correlated with the invasive score and inversely correlated with the proliferative score in both primary and metastatic melanoma samples (Fig. 4e–h).

### Functional annotation of ZEB1-AS1 correlated genes in melanoma

**Figure 5:**
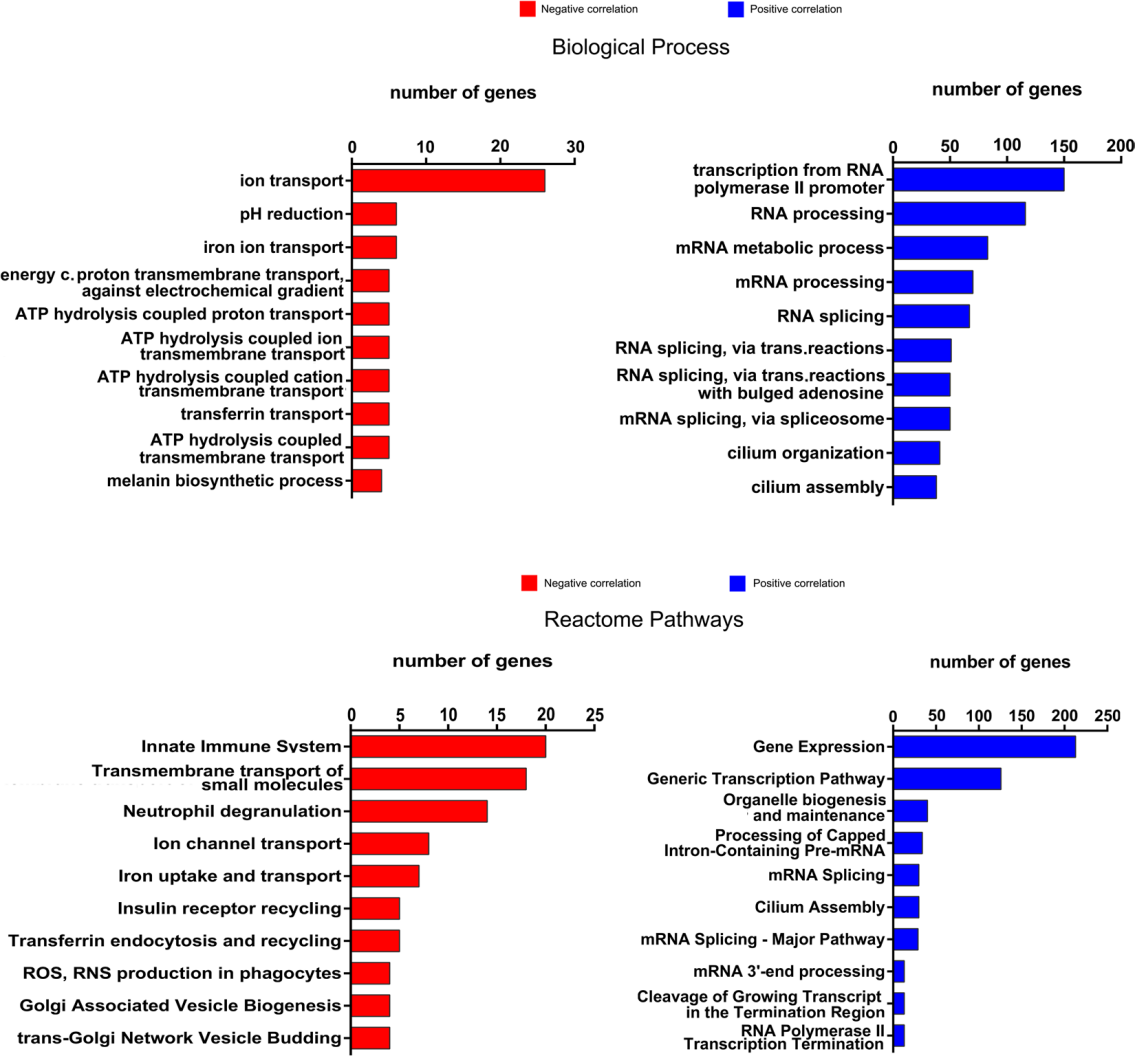
Gene Ontology (GO) and Reactome enrichment analysis showing top-10 ranked GO terms for ZEB1-AS1 correlated genes. Bar plots showing the number of significant genes for enriched Biological Process and Reactome pathways analysis, respectively. Red bars: ZEB1-AS1 negatively correlated genes (Pearson r = <−0.03). Blue bars: ZEB1-AS1 positively correlated genes (Pearson r > = 0.03).

To gain insights on the biological pathways being regulated by *ZEB1-AS1* in melanoma, we searched the RNA-Seq data from melanomas in the TCGA database for genes positively or negatively correlated with *ZEB1-AS1* expression (correlated genes). This analysis revealed 2059 Positively Correlated genes (PC, Pearson r ≥ 0.3 and *P*-value < 0.01) and 116 Negatively Correlated genes (NC, Pearson r ≤ −0.3 and *P*-value < 0.01) with *ZEB1-AS1* expression (see Supplementary Files S1 and S2). Comparatively, the same analysis for *ZEB1* gene demonstrated 1617 PC genes and 242 NC genes (Supplementary Files S3 and S4). We performed pathway enrichment analysis according to Gene Ontology (see also Supplementary Fig. S5) or Reactome annotations for the genes correlated (positively or negatively) with *ZEB1-AS1* expression. In the biological process, positively correlated genes in the top enriched pathways included transcriptional regulation, RNA processing, and cilium organization/assembly (Fig. 5). For the negative correlated genes, top-ranked biological processes were mostly associated with ion transport and intracellular pH regulation (Fig. 5).

## Discussion

In recent years, gene expression and transcriptome analysis revealed lncRNAs with aberrant expression in tumour progression and many evidences support their key role in multiple cancers^36,37^. Also, it has been demonstrated that lncRNAs present lower expression levels and are more tissue-specific than protein-coding genes, even for cancer-related lncRNAs^38–40^. We revealed that lncRNAs gene expression produces a more consistent pattern of discrimination between melanocytes and both primary and metastatic melanoma cell lines. These results demonstrate the value of lncRNA profiling as a better way to distinguish the different stages (normal melanocytes, primary tumours, and metastasis) of melanoma development.

Among the top ten upregulated lncRNAs in either primary or metastatic melanoma cell lines in comparison to melanocytes, we found *ZEB1-AS1*. Previous studies reported the involvement of *ZEB1-AS1* in different cancers, but no skin cancer associations until now^41^. *ZEB1-AS1* upregulation was first reported in hepatocellular carcinoma and associated with poor prognosis and tumour growth and metastasis^42^. Interestingly, epigenetic studies demonstrated mechanistic variance according to different tumours. *Liu and Lin* (2016), suggested that *ZEB1-AS1* directly binds and recruits the histone acetyltransferase P300 (E1A binding protein P300) to the *ZEB1* promoter region, activating *ZEB1* transcription and resulting in poor prognosis in osteosarcoma^43^. On the other hand, Su *et al*. (2017), showed that *ZEB1-AS1* binds and recruits *MLL1* (mixed lineage leukemia protein-1) to *ZEB1* promoter region, inducing H3K4me3 modification and activating *ZEB1* transcription^44^. Until now, lncRNA *ZEB1-AS1* has not been associated with melanoma and invasiveness, although there is significant evidence of *ZEB1* influence in EMT and melanoma development^27,28^. For the first time, we demonstrated lncRNA *ZEB1-AS1* upregulation in melanoma cell lines and reported significantly higher expression of this lncRNA in metastatic melanomas in comparison to primary tumours using TCGA data. In addition, we confirmed prior expectations of *ZEB1* and *ZEB1-AS1* positive correlation expression in melanoma as previously reported in some cancers^42,44^. Moreover, our analysis also increased the findings associated with this positive correlation according to normal tissues or tumours analysed (Supplementary Files S7 and S8, respectively).

Next-generation sequencing studies have identified genetic mutations that provide insights into melanoma heterogeneity, which could have implications in determining patient prognosis or targeted therapy^45–47^. Previous characterization of TCGA samples according to mutational status was utilized to identify higher expression of *ZEB1-AS1* in the *BRAF* and *RAS* mutant groups. Since both genes are found within the MAPK pathway, our findings could suggest a role of *ZEB1-AS1* in the MAPK pathway regulatory network^34^. Indeed, a recent study has suggested that resistance to MAPK inhibitors in melanoma could be due to cell plasticity mediated by high levels of *ZEB1* gene expression^48^.

As expected, by reported induction of *ZEB1* by *ZEB1-AS1*^42^, we tested well-established gene signatures associated  with an invasive or proliferative phenotype of melanomas^49^. We showed a positive correlation between higher levels of *ZEB1-AS1* and the invasive profile in melanoma cell lines and tumours. Conversely, there was a significant negative correlation with the proliferative profile score of melanocytic cells. This suggests the association of *ZEB1* and *ZEB1-AS1* with the phenotype switching in melanoma, towards the invasive phenotype. It was interesting to note that normal melanocytes demonstrated the highest scores for the proliferative profile and highest negative correlation with *ZEB-AS1*. The high proliferative score detected in cultured melanocytes is probably associated with the presence in those cells of a melanocytic cell lineage program coordinated by specific regulators such as *MITF* gene. This gene codified a transcription factor fundamental to differentiation and development of melanocytic cell lineage and associated with the proliferative profile of melanoma cells^50^. In this sense, the crucial role of *MITF* besides determining embryonic melanocyte fate includes regulation of downstream genes involved in cell cycle progression such as *CDK2*^51^ (cyclin-dependent kinase 2) and cell proliferation inducers as *DIAPH1*^52^(diaphanous related formin 1). In fact, it is suggested that *MITF* regulatory potential in normal endogenous levels stimulate proliferation while down or overexpression inhibits cell proliferation competence^53^.

Although the role of *ZEB1*, and other key transcription factors, in promoting invasion and malignant progression in many cancers^54–56^ is well established, associations of lncRNAs and invasiveness and tumour development still need more research. We observed a positive association between *ZEB1-AS1* expression with the invasive phenotype in melanoma tumours. In addition, *ZEB1-AS1* expression levels positively correlated with several genes involved in transcription regulation, indicative of a role in transcriptional reprogramming, a central process for phenotype switching. An example of a gene in this pathway is the transcription factor *MZF1* (myeloid zinc finger 1) gene. This gene is known to contribute to malignancy and invasion in many tumours^57^. Indeed, it has been recently associated with *PRAME* (preferentially expressed antigen of melanoma) upregulation and colony formation, which is associated with metastatic potential in melanoma cell line studies^58^. Also, interestingly, genes involved in cilium formation were considered enriched among the genes positively correlated with *ZEB1-AS1* in melanomas. Although the loss of primary cilia has been more frequently implicated in the development of melanoma^59^ and other cancers^60^, a recent study unveiled primary ciliogenesis as a key mechanism by which EMT programs induce stemness in a specific subset (claudin-low subtype) of breast tumours^61^. In this sense, it would be interesting to investigate whether *ZEB1-AS1* has any role in the regulation of primary cilium formation and if this regulation may affect melanoma development and invasion in general or in a subset of melanoma tumours.

The association of *ZEB1-AS1* expression with melanoma invasion profile is certainly dependent in part of *ZEB1* function. However, our analysis pointed out also for a *ZEB1* independent role of this lncRNA in the transcriptional regulation, since 82,1% of the positively and 25% of the negatively correlated genes with *ZEB1-AS1* were not shared with *ZEB1*. Despite *ZEB1* gene being known as a transcriptional repressor, our data analysis supports *ZEB-AS1* as a positive modulator of gene expression^62^, like some antisense lncRNAs have been described to act like^63^.

In summary, we firstly identified lncRNA *ZEB1-AS1* upregulation in melanoma when compared with melanocytes and metastatic tumours in comparison to primary melanomas. In addition, we showed an association between *ZEB1-AS1* expression levels with *BRAF*/*RAS* status and with an invasive phenotype in melanoma and suggested this lncRNA directly or indirectly regulates potential genes and pathways. These findings support the notion of an important function of *ZEB1-AS1*, which may act with some independence of *ZEB1*, in melanoma progression and melanoma phenotype switching. To this date, lncRNAs interactions with diverse molecules have proved to be an essential aspect as modulators of cancer phenotypes^64^. Altogether, these evidences and our findings reinforce the need for further functional analysis, including the characterization of mechanistically associated molecules, of *ZEB1-AS1* in melanoma progression.

## Methods

### Cell culture

Primary melanoma (WM35, WM1552, WM278, WM793 and WM1789) and metastatic melanoma (1205lu, WM1617 and WM852) cell lines were grown in conditions of 5% CO_2_ atmosphere and temperature of 37 °C. These melanoma cell lines were maintained in TU growth medium (80% of MCDB-153 medium, 20% of Leibovitz’s L-15 medium, 5 µg/mL of Insulin and 2 mM of CaCl_2_) supplemented with 2% Fetal Bovine Serum (Gibco) as previously described^65^, until harvesting at 80% of plate confluence. Dr. Meenhard Herlyn at the Wistar Institute (Philadelphia, PA) kindly provided all melanoma cell lines. Primary cultures of skin cells (melanocytes – FM308) were obtained from the foreskins of University Hospital (Hospital Universitário – HU-USP) patients, donated by Dr. Linda Maximiano. To this end, the project has undergone review and approval by the Ethics Committee of HU (HU no. CEP Case 943/09) coordinated by Dr. Silvya Stuchi Maria-Engler. Melanocytes were grown in medium 254CF (Thermo Fischer Scientific), supplemented with HMGS solution (Thermo Fischer Scientific) and 200 µM calcium chloride and maintained as previously described^66,67^. All cell lines utilized in this work were tested for mycoplasma infection.

### RNA extraction

Total RNA was extracted with AllPrep DNA/RNA/miRNA Universal Kit (Qiagen) following the manufacturer’s instructions. Density and purity of RNA were tested by the ratio 260/280 nm measurement in NanoDrop (Thermo Fischer Scientific). The RNA quality was tested by Agilent Bioanalyzer, which generates an RNA Integrity Number (RIN) between 1 (more degraded) and 10 (least degraded) and only samples with threshold RIN ≥7 were utilized in this study.

### Library construction and RNA sequencing

RNA fragments sized 200–500 bp (base pairs) obtained by fragmentation were utilized to generate sequencing libraries using TruSeq Stranded Total RNA LT Sample Prep Kit (Illumina Inc). The RiboZero technology (Illumina Inc) was utilized to remove abundant rRNA and keep both poly(A) and non-poly(A) transcripts. Clustering was made in an automated system cBot (Illumina Inc), and samples were sequenced with TruSeq SBS kit v5, single-read 72 cycles in Genome Analyzer IIx equipment (Illumina Inc). All reagents were utilized following manufacturer’s usage protocols.

### Public raw data and pre-processing

Raw sequencing data from melanocyte (MC502), primary melanoma (A375) and metastatic melanoma cell lines (A2058, C32, MALME3M, SKMEL28, SKMEL5, and WM2664) were downloaded from Gene Expression Omnibus (GEO), under access number GSE46818. Then, the data was converted to compatible *fastq* format file using the SRA Toolkit (http://www.ncbi.nlm.nih.gov/Traces/sra/sra.cgi?view=toolkit_doc&f=fastq-dump), and quality control was tested using FastQC (Babraham Bioinformatics).

### Transcriptome mapping, annotation, and quantification

All data were mapped and quantified by STAR v2.5^68^. We considered a count feature as a count read per gene while mapping, as package default parameters from STAR, and quantifications were displayed as CPM (Counts Per Million) for cell lines. The lncRNA gene annotations were downloaded from GENCODE Release 25 (GRCh38). Therefore, different biotypes of lncRNAs were categorized according to those provided from the long noncoding genes annotation in the GTF file.

### Data normalization and differential gene expression analysis

Public and in house-generated RNA-Seq data were normalized using the default method Trimmed Mean of M-values (TMM) to avoid bias effects. To detect differentially expressed lncRNAs, edgeR package version 3.16.5 from Bioconductor in R statistical platform was used. We considered differentially expressed genes in analysis with adjusted *P*-values <  0.05 and Fold Change (FC) ≤−2 or ≥2 for down and up-regulated genes, respectively.

### TCGA data analysis and mutational classification of melanoma samples

TCGA gene expression data was downloaded directly from the TCGA portal and via UCSC repository (xenabrowser.net). TCGA samples were matched by barcode and divided into four main groups according to the presence or not of mutations established by genomic molecular classification of melanoma provided by TCGA team^34^. This classification relied on the division of SKCM according to the presence of melanoma hotspot mutations (*BRAF*, *NF1,* and *RAS* family group) or their absence (Triple Wild-Type).

### Gene Set Variation Analysis and melanoma proliferative/invasive gene expression signatures

Gene Set Variation Analysis (GSVA)^69^ allowed us to define an enrichment score (ES) that represents the degree of enrichment of a gene set in each sample within a given dataset^70^. Based on the systematic classification of melanoma by phenotype-specific gene expression profile^49^, we retrieved melanoma gene expression signatures associated with either invasive (n = 45 genes, Supplementary File S5) or proliferative status (n = 52 genes, Supplementary File S6). Accordingly, we evaluated the correlation between either *ZEB1* or *ZEB1-AS1* expression with the proliferative or invasive ES presented by melanoma cell lines and TCGA melanoma tumours.

### Correlated genes, Gene Ontology, and KEGG analysis

Pearson’s correlation coefficient was calculated between lncRNA *ZEB1-AS1* and each other gene in melanoma samples from the TCGA. Genes significantly correlated (r ≤ −0.3 or r ≥ 0.3 and *P*-value < 0.01) were submitted to the functional annotation in Gene Ontology (GO) terms for biological processes (BP), cellular components (CC), and molecular function (MF). Then, top-ranked GO terms were displayed according to the highest number of genes assigned. Positive and negative *ZEB1-AS1* correlated genes were also submitted to pathway enrichment analysis using the Reactome database. Both analyses were performed using the WEB-based Gene SeT AnaLysis Toolkit (WebGestalt)^71^.

### Statistical analysis

Statistical tests and plots were calculated and generated using R platform and PRISM V.6.01 (GraphPad Software, La Jolla California USA).

## Supplementary information

Supplementary Information

Supplementary File S1

Supplementary File S2

Supplementary File S3

Supplementary File S4

Supplementary File S5

Supplementary File S6

Supplementary File S7

Supplementary File S8

## Data Availability

The datasets generated during the current study are available in the SRA database repository, under SRA accession PRJNA530784 (http://www.ncbi.nlm.nih.gov/sra/PRJNA530784).
