# Supplementary material for: Whole transcriptome analysis reveals correlation of long noncoding RNA ZEB1-AS1 with invasive profile in melanoma
Source: Sci Rep. 2019 Aug 5;9:11350. doi: 10.1038/s41598-019-47363-6 (PMC6683136; doi:10.1038/s41598-019-47363-6)
Supplement: Supplementary file 1 — Supplementary Information [file 41598_2019_47363_MOESM1_ESM.pdf]

SUPPLEMENTARY INFORMATION

Whole transcriptome analysis reveals correlation of long noncoding RNA ZEB1-AS1 with invasive profile in melanoma

Ádamo Davi Diógenes Siena[1,3], Jéssica Rodrigues Plaça[3,4], Luiza Ferreira Araújo[1,3,4], Isabela Ichihara de Barros[1,3], Kamila Peronni[3], Greice Molfetta[1,3,6], Carlos Alberto Oliveira de Biagi Júnior[1,3], Enilza Maria Espreafico[2], Josane Freitas Sousa[3,4,5], Wilson Araújo Silva Jr[1,3,4,6] *

[1]Department of Genetics at Ribeirão Preto Medical School, University of São Paulo, Ribeirão Preto, Brazil.

[2]Department of Cellular and Molecular Biology at Ribeirão Preto Medical School, University of São Paulo, Ribeirão Preto, Brazil.

[3]Center for Cell-Based Therapy (CEPID/FAPESP); National institute of Science and Technology in Stem Cell and Cell Therapy (INCTC/CNPq), Regional Blood Center of Ribeirão Preto, Ribeirão Preto, Brazil.

[4]Center for Integrative Systems Biology (CISBi) – NAP/USP. Ribeirão Preto, Brazil.

[5]Institute of Biological Sciences, Federal University of Para, Belem, Brazil.

[6]Center for Medical Genomics, HCFMRP/USP, Ribeirão Preto, Brazil.

*Corresponding author

E-mail address: wilsonjr@usp.br

Supplementary Figure S1.

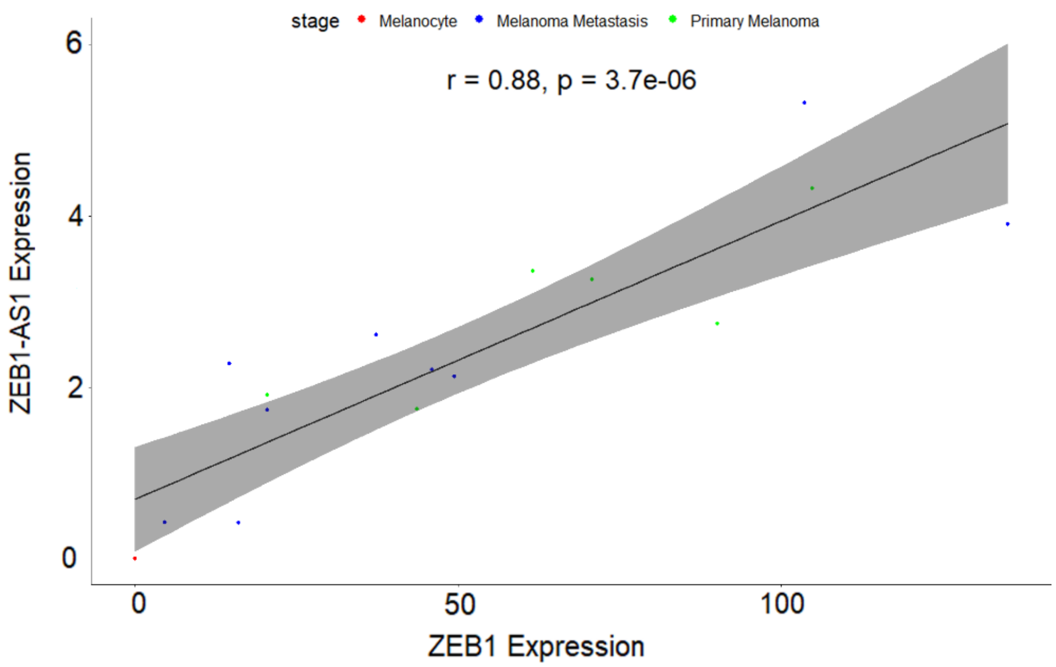

Supplementary Figure S1. Analysis of correlation between ZEB1-AS1 and ZEB1 gene expression levels in melanocyte and melanoma cell lines.

Supplementary Figure S2.

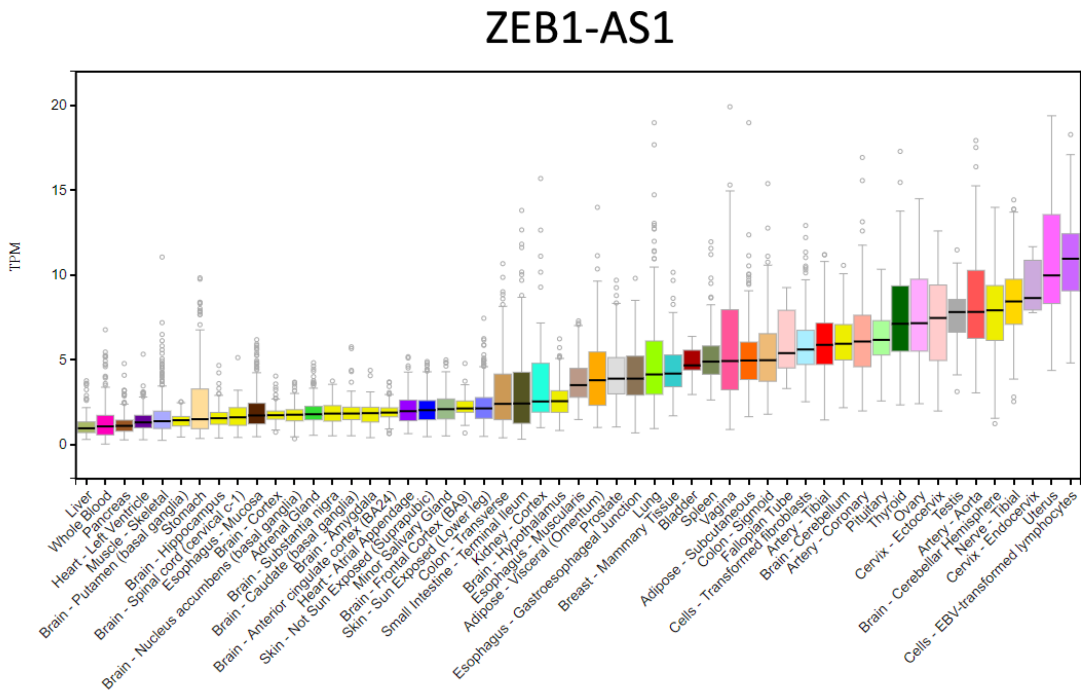

Supplementary Figure S2. Analysis of ZEB1-AS1 gene expression in 53 different normal tissues from GTEx consortium.

Supplementary Figure S3.

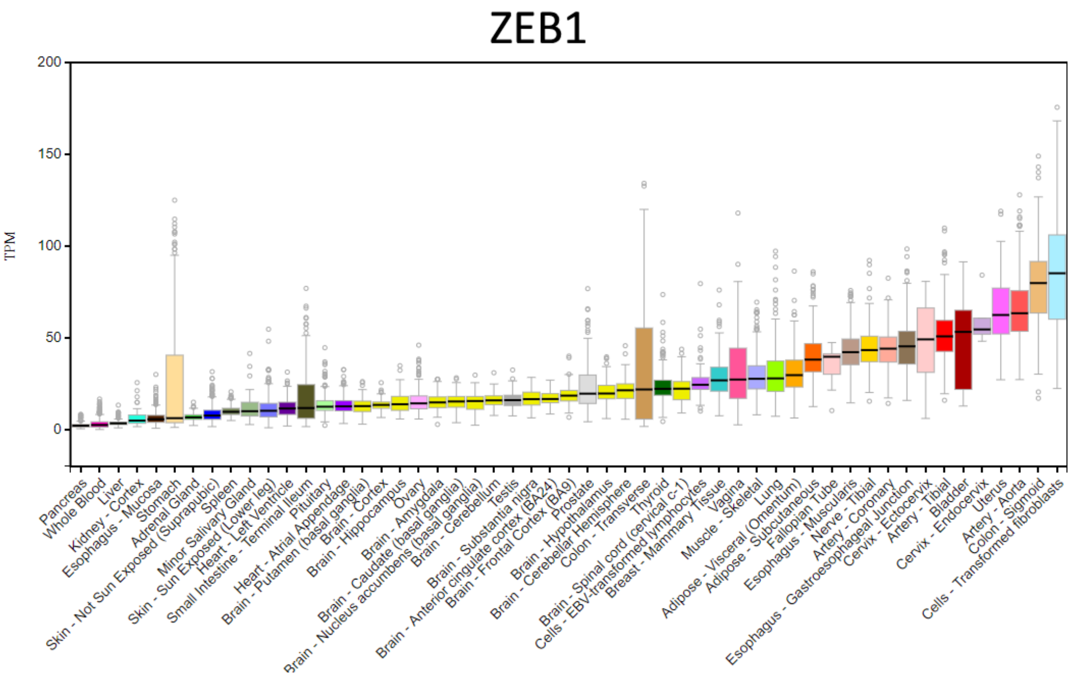

Supplementary Figure S3. Analysis of ZEB1 gene expression in 53 different normal tissues from GTEx consortium.

Supplementary Figure S4.

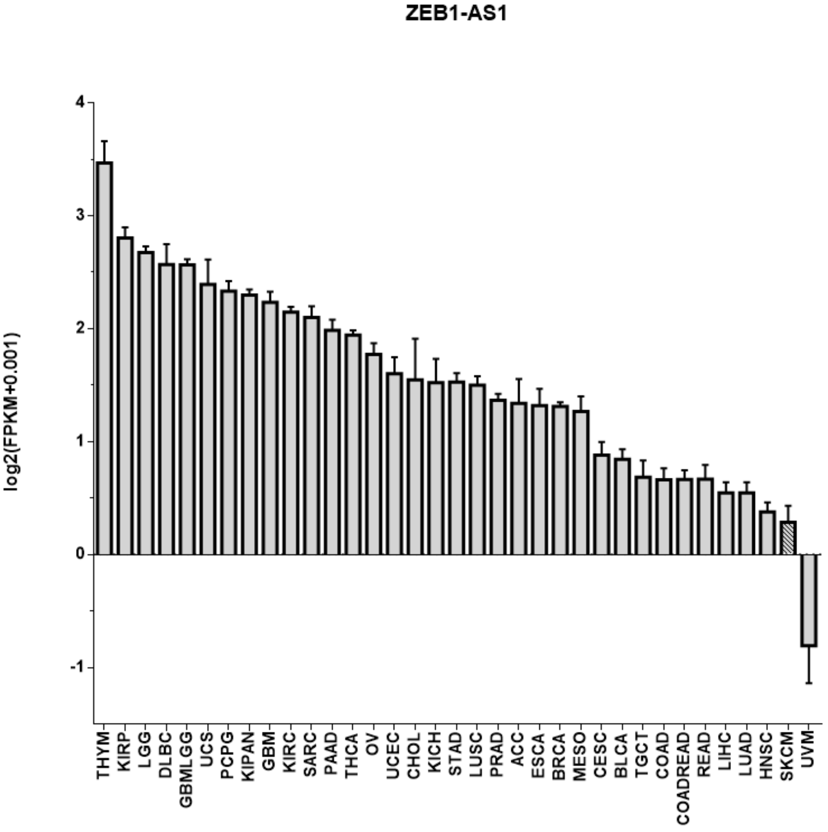

Supplementary Figure S4. The ZEB1-AS1 lncRNA gene expression in different cancers. Analysis of ZEB1-AS1 gene expression in 32 different tumor samples from TCGA. The hatched bar highlights ZEB1-AS1 in cutaneous melanoma (SKCM). ACC (Adrenocortical carcinoma), BLCA (Bladder Urothelial Carcinoma), BRCA (Breast invasive carcinoma), CESC (Cervical squamous cell carcinoma and endocervical adenocarcinoma), CHOL (Cholangiocarcinoma), COAD (Colon adenocarcinoma), DLBC (Lymphoid Neoplasm Diffuse Large B-cell Lymphoma), ESCA (Esophageal carcinoma), GBM (Glioblastoma multiforme), HNSC (Head and Neck squamous cell carcinoma), KICH (Kidney Chromophobe), KIRC (Kidney renal clear cell carcinoma), KIRP (Kidney renal papillary cell carcinoma), LGG (Brain Lower Grade Glioma), LIHC (Liver hepatocellular carcinoma), LUAD (Lung adenocarcinoma), LUSC (Lung squamous cell carcinoma), MESO (Mesothelioma), OV (Ovarian serous cystadenocarcinoma), PAAD (Pancreatic adenocarcinoma), PCPG (Pheochromocytoma and Paraganglioma), PRAD (Prostate adenocarcinoma), READ (Rectum adenocarcinoma), SARC (Sarcoma), STAD (Stomach adenocarcinoma), TGCT (Testicular Germ Cell Tumors), THCA (Thyroid carcinoma), THYM (Thymoma), UCEC (Uterine Corpus Endometrial Carcinoma), UCS (Uterine Carcinosarcoma), UVM (Uveal Melanoma).

Supplementary Figure S5.

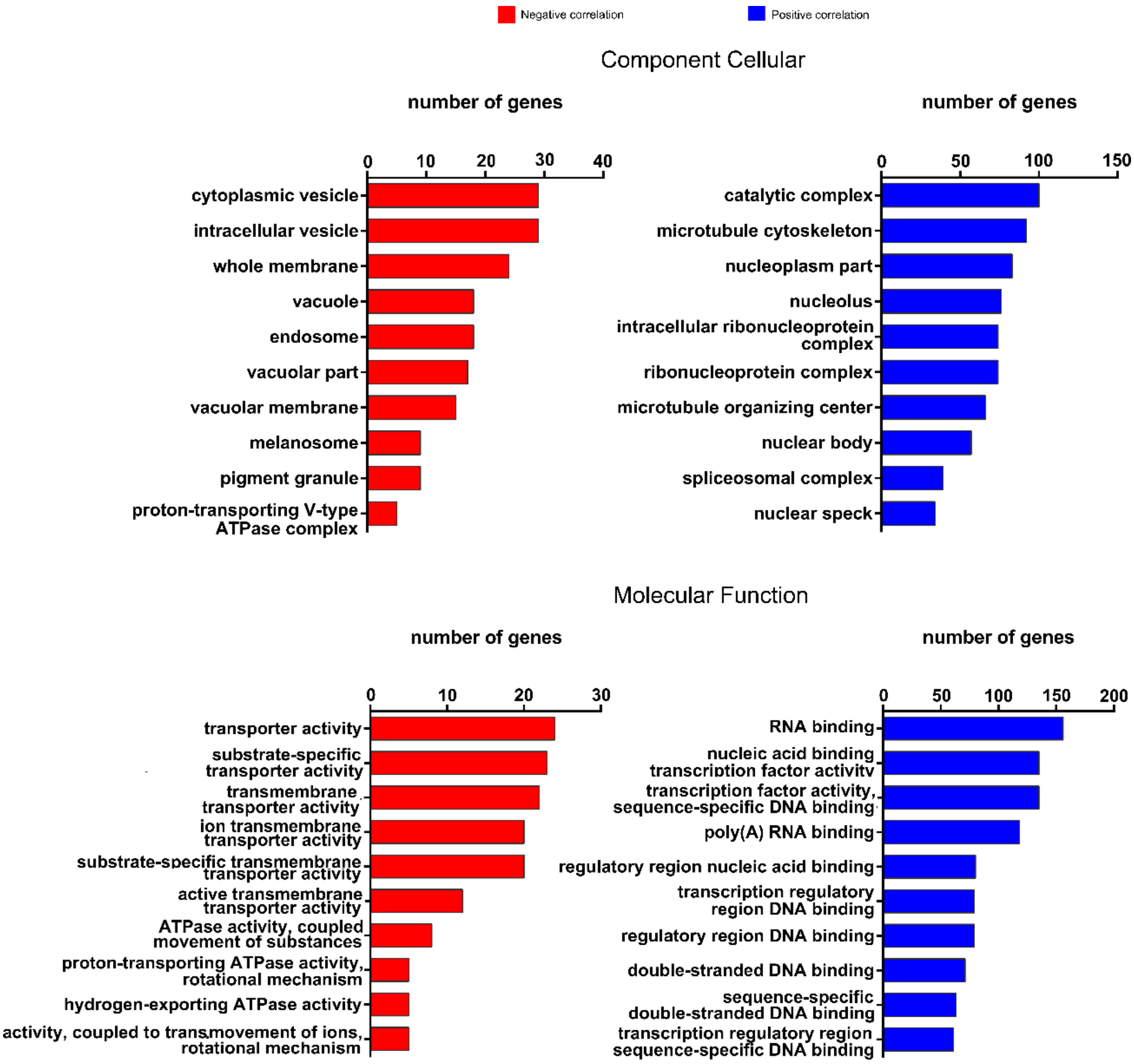

Supplementary Figure S5. Gene ontology analysis for component cellular and molecular function terms showing top-10 ranked terms for positively correlated genes (blue bars) and negatively correlated genes (red bars).

Supplementary Table S1.

	1205LU	FM308	MC502	A2058	A375	C32	MalMe3M	SKMEL28	SKMEL5	WM2664	WM1552	WM1617	WM1789	WM278	WM35	WM793	WM852	Mean (%)
Number of input reads	60858294	56615826	30745266	39780187	39470556	26581030	34827767	37168617	31433323	31865511	58453123	60867353	72474390	80751546	55502396	53753191	48128159	
Uniquely mapped reads number	38766391	45497938	27996114	29707887	31788663	16923338	28169920	29141556	22694112	22638402	51490890	18775634	21775594	41570304	47700610	20263339	21257948	
Uniquely mapped reads %	63,7	80,36	91,06	74,68	80,54	63,67	80,88	78,4	72,2	71,04	88,09	30,85	30,05	51,48	85,94	37,7	44,17	64,39

Supplementary Table S1. Average mapping efficiency from cell lines of in-house generated RNA-Seq and public available data.
